# Cortical aging – new insights with multiparametric quantitative MRI

**DOI:** 10.18632/aging.103629

**Published:** 2020-08-27

**Authors:** Alexander Seiler, Sophie Schöngrundner, Benjamin Stock, Ulrike Nöth, Elke Hattingen, Helmuth Steinmetz, Johannes C. Klein, Simon Baudrexel, Marlies Wagner, Ralf Deichmann, René-Maxime Gracien

**Affiliations:** 1 Department of Neurology, Goethe University, Frankfurt am Main, Germany; 2 Department of Neuroradiology, Goethe University, Frankfurt am Main, Germany; 3 Brain Imaging Center, Goethe University, Frankfurt am Main, Germany; 4 Nuffield Department of Clinical Neurosciences, University of Oxford, Oxford, UK

**Keywords:** cortical aging, tissue microstructure, quantitative MRI, surface-based analysis, iron deposition

## Abstract

Understanding the microstructural changes related to physiological aging of the cerebral cortex is pivotal to differentiate healthy aging from neurodegenerative processes. The aim of this study was to investigate the age-related global changes of cortical microstructure and regional patterns using multiparametric quantitative MRI (qMRI) in healthy subjects with a wide age range. 40 healthy participants (age range: 2^nd^ to 8^th^ decade) underwent high-resolution qMRI including T1, PD as well as T2, T2* and T2′ mapping at 3 Tesla. Cortical reconstruction was performed with the FreeSurfer toolbox, followed by tests for correlations between qMRI parameters and age. Cortical T1 values were negatively correlated with age (p=0.007) and there was a widespread age-related decrease of cortical T1 involving the frontal and the parietotemporal cortex, while T2 was correlated positively with age, both in frontoparietal areas and globally (p=0.004). Cortical T2′ values showed the most widespread associations across the cortex and strongest correlation with age (r= -0.724, p=0.0001). PD and T2* did not correlate with age. Multiparametric qMRI allows to characterize cortical aging, unveiling parameter-specific patterns. Quantitative T2′ mapping seems to be a promising imaging biomarker of cortical age-related changes, suggesting that global cortical iron deposition is a prominent process in healthy aging.

## INTRODUCTION

As a consequence of a constantly increasing life expectancy in our societies, the investigation of human brain aging has emerged as an important and growing research field in neuroscience [1]. The process of biological aging is not only dependent on chronological ageing [2], as it is widely acknowledged that certain lifestyle habits and comorbidities such as cardiovascular and metabolic disorders may favor and accelerate aging processes in the brain [2–5]. The cerebral cortex is generally assumed to play a key role in cognitive abilities in higher age groups. In particular, cognitive deficits in old age predominantly involve long-term and working memory as well as the ability to form new episodic memories [6] and thus functions with assumed cortical representation. Gaining a deeper understanding of healthy cortical ageing is fundamental for elucidating the transition between normal aging and prodromal stages of pathological neurodegeneration, as well as the interactions between ageing and additional detrimental effects of diseases on the cortical microstructure.

Previous studies assessing the cerebral cortex with conventional magnetic resonance imaging (MRI) techniques reported age-related reductions in cortical thickness [7, 8], cortical volume [9] and the cortical surface area [10]. In addition, specific regional patterns of these cortical gray matter (GM) changes have been demonstrated [7, 10–12], indicating locally varying vulnerability or resistance to age-related involutional alterations of tissue structure. However, conventional MRI techniques yield images with mixed contrasts and are therefore not fully suited to provide information on the microstructural processes that underlie and potentially precede cortical atrophy.

In contrast, quantitative MRI (qMRI) allows for assessing subtle changes in tissue microstructure [13], measuring a variety of physical parameters which are specifically related to certain tissue properties associated with pathological or age-driven changes of the microstructural tissue composition [14]. Since different distinct processes on the microstructural level such as involution of the neuropil, demyelination, iron accumulation and changes of tissue water content are associated with aging [15–19], qMRI may provide more profound insights into the respective microstructural tissue changes. Previous studies described age-related changes of qMRI parameters, suggesting changes in cortical tissue properties, such as the degree of myelination and the iron and water fraction, caused by physiological aging [20–28]. For instance, several studies congruently reported a decrease of cortical T1 relaxation time [23–27] and an increase of cortical T2 relaxation time [24, 25, 28] during the entire adulthood in healthy aging. However, comprehensive multi-parametric analyses of age-related changes of cortical qMRI parameters combined with an investigation of their spatial distribution across the cortex and a comparison of the spatial patterns between parameters might allow for a deeper understanding of natural cortical aging.

In this cross-sectional study, we used qMRI techniques measuring the longitudinal relaxation time (T1), the proton density (PD) and the irreversible, effective and reversible transverse relaxation times (T2, T2* and T2′) in combination with surface-based analyses to investigate cortical patterns of the dependence between the cortical microstructure and age in a group of adult healthy volunteers with a wide age range. We hypothesized to observe differences, both in the spatial distribution and the extent of different age-associated microstructural processes such as changes of the myelin, water and iron proportions, and of the corresponding changes of qMRI parameters across the cerebral cortex. Since a previous study using positron emission tomography (PET) has demonstrated a decline of aerobic glycolysis with increasing age in frontoparietal and upper temporal regions [29], we assumed that age-related changes of tissue microstructure might be pronounced in these cortical areas. Furthermore, as cerebral iron deposition is considered an important age-related process involving various brain structures [30], we hypothesized that an increasing iron content might be a prominent process in cortical aging.

## RESULTS

The mean age ± standard deviation (SD) of the included subjects was 38.8 ± 14.97 years (range: 19-71 years, 2^nd^ to 8^th^ decade), with 19 male (47.5 %) and 21 female (52.5 %) participants.

### The relationship between age and mean qMRI parameters/cortical thickness across the cerebral cortex

**Figure 1:**
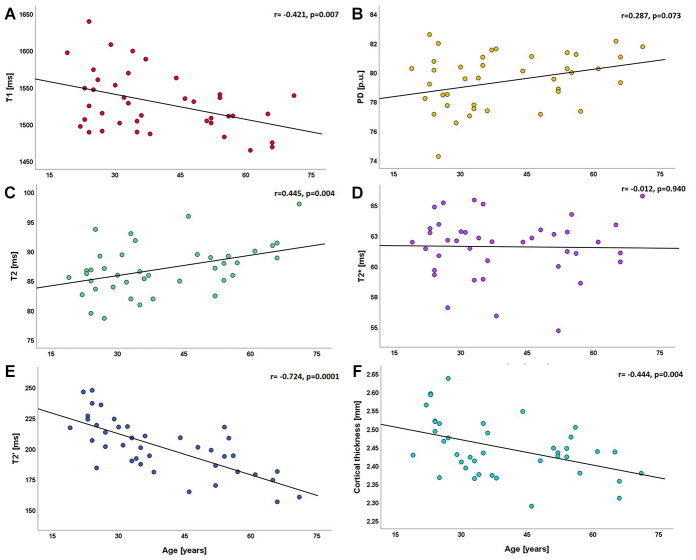
**Scatterplots illustrating the relationship between global cortical qMRI parameters/cortical thickness and age.** (**A**) relationship between T1 and age; (**B**) relationship between PD and age; (**C**–**E**) relationships of T2, T2* and T2' with age; (**F**) relationship between cortical thickness and age. ms: milliseconds; p.u.: percentage units; mm: millimeters.

Global quantitative T1 values were significantly negatively correlated with age (r= -0.421, p=0.007, Figure 1A), while for PD values a significant relationship with age could not be detected (r=0.287, p=0.073, Figure 1B). Quantitative T2 values across the entire cortex showed a strong positive correlation with age (r=0.445, p=0.004, Figure 1C). For T2* values, no significant correlation with age was found on a global level (r= -0.012, p=0.940, Figure 1D). There was a strong negative correlation between whole-cortex quantitative T2′ values and age (r= -0.724, p=0.0001, Figure 1E). Similarly, the global cortical thickness was strongly negatively correlated with age (r= -0.444, p=0.004, Figure 1F). For each parameter, global mean values and standard deviations (SD) across the cortex (serving as a measure of the cortical distribution) are provided in Table 1. For graphical illustration, Supplementary Figure 1 shows the statistical spread across the group for each SD.

**Table 1:** Distribution of the parameter values: mean values and standard deviations across the cortex.

**Parameter**	**Mean across cortex**	**SD across cortex**
T1 [ms]	1528.75 ± 40.58	146.73 ± 9.55
PD [p.u.]	79.48 ± 1.85	5.45 ± 0.46
T2 [ms]	86.99 ± 4.17	27.69 ± 8.66
T2* [ms]	61.60 ± 2.48	43.37 ± 9.00
T2′ [ms]	201.50 ± 22.47	90.83 ± 7.39
Cortical thickness [mm]	2.45 ± 0.08	0.62 ± 0.03

The mean ± the standard deviation across the group are presented for each parameter. ms: milliseconds; p.u.: percentage units; mm: millimeters; SD: standard deviation. For a detailed illustration of the distribution of the standard deviations across the cortex, please see the Supplementary Figure 1.

After controlling for the total intracranial volume (TIV) and sex as confounding variables by partial correlations, similar associations between qMRI parameters across the entire cortex and age were detected: quantitative T1 values showed a significant negative correlation with age (r= -0.421, p=0.009), while irreversible (T2) and reversible (T2′) quantitative transverse relaxation times showed significant correlations in diverging directions (T2: r=0.440, p=0.006 and T2′: r= -0.722, p=0.0001). There was globally no significant correlation between quantitative T2* (r= -0.030, p=0.856) or PD (r=0.306, p=0.062) values and age. Analysis of the association between global cortical qMRI parameters and global cortical thickness (controlling for the effect of the TIV) revealed a significant negative correlation between global cortical T2 and cortical thickness (r= -0.424, p=0.007), while global cortical T2′ values correlated positively with cortical thickness (r=0.647, p=0.0001). Global cortical T2* and PD values were not significantly correlated with cortical thickness (r= -0.029, p=0.861 and r=0.116, p=0.481). Among all qMRI parameters that were globally correlated with age, only cortical T1 values did not show a relationship with cortical thickness (r= -0.021, p=0.898).

### Patterns and regional distributions of age-related changes in cortical qMRI parameters and cortical thickness

**Figure 2:**
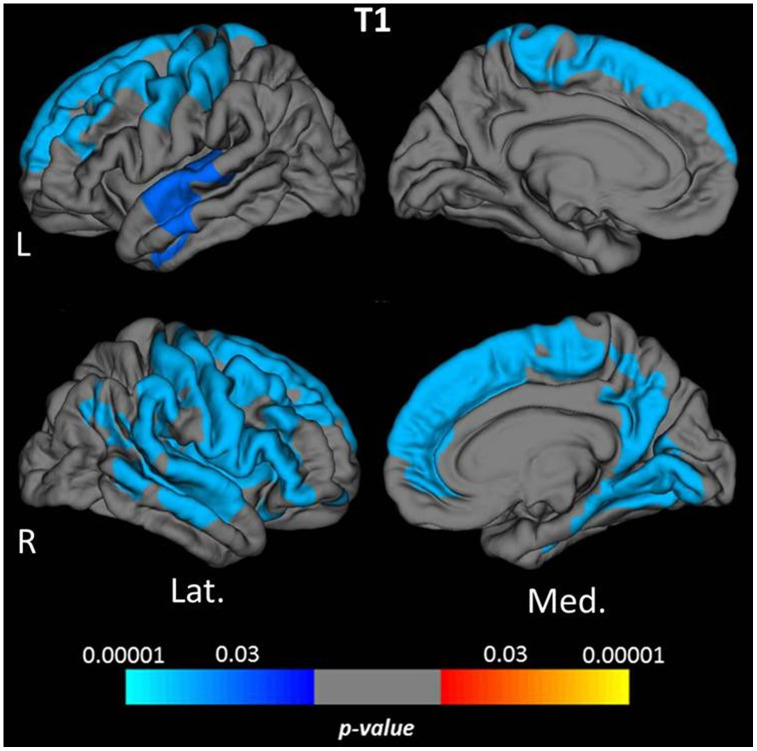
**Cortical clusters indicating a significant association between age and cortical quantitative T1 values.** The scale bar displays the level of significance. Cold colors demonstrate a negative association with age in the respective regions. L: left; R: right; Lat.: lateral; Med.: medial.

**Figure 3:**
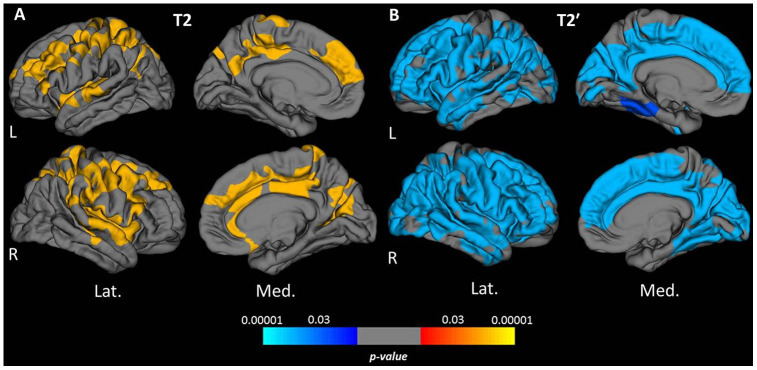
****Cortical clusters exhibiting a significant relationship between age and cortical quantitative T2 (**A**) and T2′ values (**B**). The scale bar displays the level of significance. Hot colors demonstrate a positive and cold colors a negative association with age in the respective regions. L: left; R: right; Lat.: lateral; Med.: medial.

**Figure 4:**
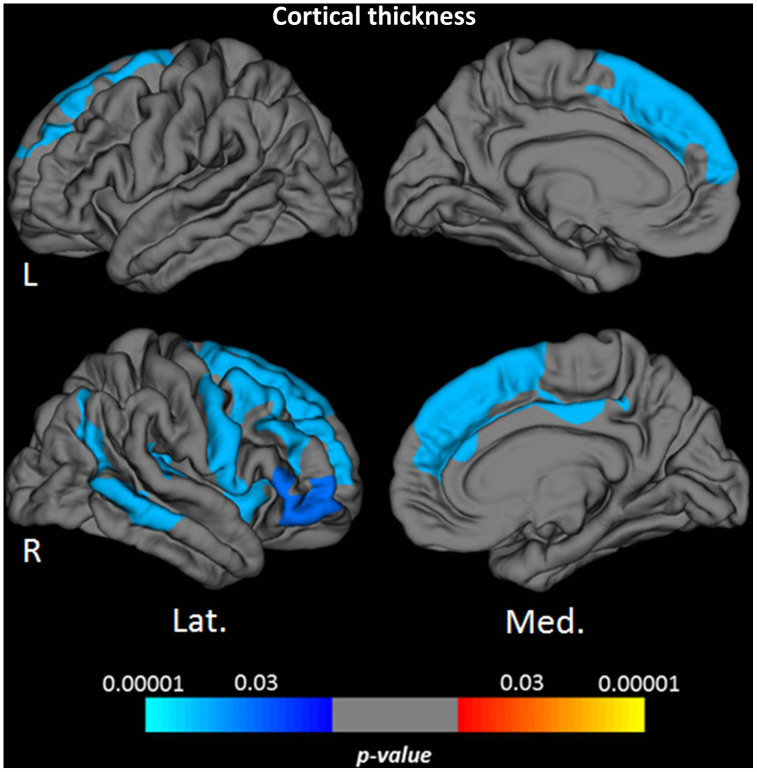
**Cortical clusters indicating a significant relationship between age and cortical thickness.** The scale bar indicates the level of significance. Cold colors demonstrate a negative association with age in the respective regions. L: left; R: right; Lat.: lateral; Med.: medial.

Vertex-wise surface-based analysis of cortical qMRI parameters, conducted for the parameters which globally showed significant associations with age, revealed distinct patterns and regional distributions of clusters of surface-points/vertices, showing a significant association with age (Figures 2 and 3, warm/cold colors representing a positive/negative correlation of the respective parameter with age). Cortical regions with a significant negative association of quantitative T1 values and age were mainly located in dorsofrontal as well as in the upper temporal regions (Figure 2) for both hemispheres. In the right hemisphere, regions with a significant relationship of T1 and age were also located in the medial parietal, temporal and occipital cortex (Figure 2). Age-related increases of cortical quantitative T2 values showed a similar pattern as compared to decreases of cortical T1, however, with smaller clusters located in the upper frontal cortex, the upper temporal cortex and the right medial parieto-occipital cortex (Figures 2, 3A). The reversible transverse relaxation time T2′ showed widespread significant negative associations with age in clusters involving large parts of the cortex with similar patterns of quantitative T2′ changes for both hemispheres (Figure 3B). Significant correlations between cortical thickness and age were mainly found in the upper and medial frontal cortex in both hemispheres as well as in frontobasal and temporoparietal regions of the right hemisphere (Figure 4). Since the numerous small clusters indicating a significant association between cortical thickness and age observed for the left lateral hemisphere before correction for multiple comparisons were susceptible to the correction, resulting in a substantial change of the original pattern, an additional figure (Supplementary Figure 2) shows the respective uncorrected map for the left lateral hemisphere, demonstrating more widespread associations with age.

**Figure 5:**
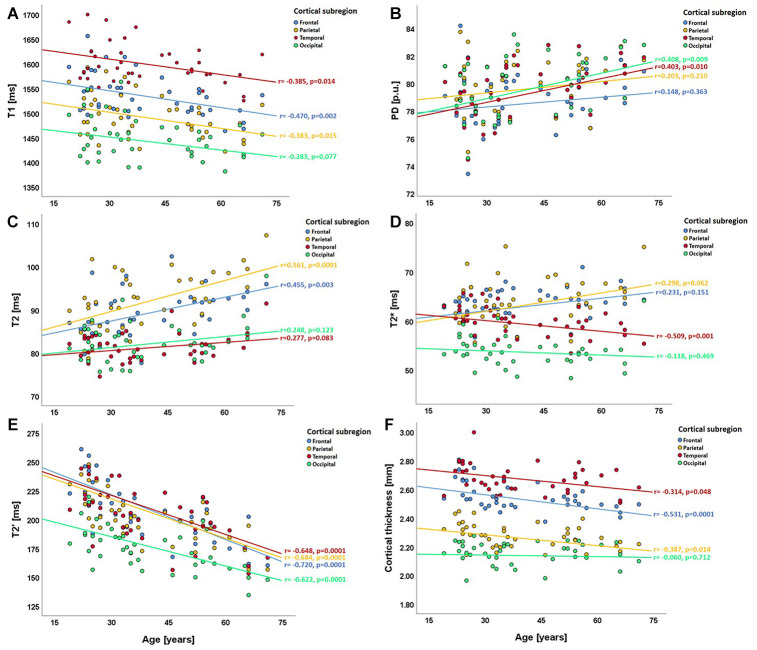
**Scatterplots illustrating the relationship between regional cortical qMRI parameters/cortical thickness and age in the cortical subregions (lobes).** (**A**) relationship between T1 and age; (**B**) relationship between PD and age; (**C**–**E**) relationships of T2, T2* and T2' with age; (**F**) relationship between cortical thickness and age. In each plot, the four different colors denote the respective cortical subregions. ms: milliseconds; p.u.: percentage units; mm: millimeters.

Regional analysis of qMRI values in the cortical lobes revealed a consistent negative association between cortical T1 values and age, which, however, missed statistical significance in the occipital cortex (Figure 5A). PD values showed weak to moderate correlations with age, reaching statistical significance in the temporal and the occipital cortex (Figure 5B). While cortical T2 values showed positive correlations with age, which were statistically significant in the frontal and partial lobes (Figure 5C), a strong negative correlation with age was observed for T2′, which was statistically significant throughout all cortical subregions (Figure 5E). In contrast, cortical T2* values exhibited a regionally heterogeneous relationship with age (Figure 5D), attaining statistical significance only in the temporal cortex. The negative correlation between cortical thickness and age was most pronounced in the frontal and parietal cortex (Figure 5F).

## DISCUSSION

This study investigated the relationship between cortical qMRI parameters and age via a surface-based analysis approach, detecting the spatial distributions of changes in cortical microstructure related to increasing age with focus on the 2^nd^ to the 8^th^ decade.

Quantitative T2′ values showed a strong negative correlation with age and the most widespread age-related changes among all investigated qMRI parameters (Figure 3B). This result suggests high sensitivity of T2′ mapping to microstructural changes underlying normal cortical aging. The observed T2′ shortening with increasing age is in line with the results of a previous study describing age-related changes of cortical T2′ [31]. However, the presented study allows for a comparison of the relationship between T2′ and age with the age-dependence of other parameters and of the respective spatial distributions. Quantitative T2′ values resulting from a correction of the effective transverse relaxation time T2* for spin-spin effects have been proposed as a sensitive measure for the estimation of relative parenchymal iron content [32, 33]. In concordance with results from histopathological and qMRI studies indicating age-related cortical iron deposition [19, 34–38], the observed decrease of cortical T2′ values in the presented analysis (Figures 1E, 3B, 5E) suggests a considerable increase of cortical tissue iron levels across the adult life span caused by physiological aging.

The obtained mean cortical T2 values were positively correlated with age and clusters indicating a significant association with age were mainly located in the frontoparietal and upper temporal cortex (Figures 1C, 3A, 5C). The increase of cortical T2 values with higher age is in agreement with previous studies [24, 25, 28]. Conceivable changes of cortical microstructure with an effect on T2 that are known to occur in healthy ageing primarily include demyelination (causing an increase of T2) [39, 40] and increase of tissue iron content (leading to a decrease of T2) [30, 35, 41]. Furthermore, a prolongation of the T2 relaxation time can be caused by increased tissue water content [21, 42, 43] and gliotic tissue conversion [43]. As revealed by the analysis of cortical T2′, iron deposition most likely plays an important role in cortical aging. Yet, processes leading to T2 increases, especially myelin loss [16], seem to have a stronger impact on cortical T2 in normal aging. A shrinking of the neuronal volume fraction and relative increase of the glial volume fraction, as described in histopathological studies [44–47], could also potentially contribute to the prolongation of cortical T2. For cortical T2′ values, which showed widespread negative associations with age across the cortex (Figures 3B, 5E) and for cortical T2 values (Figures 3A, 5C) the results of the analysis based on cortical subregions was in line with the results of the vertex-wise analysis.

Global cortical T2* values did not show a significant relationship with age (Figure 1D) and the regional analysis only revealed significant negative correlation with age in the temporal lobe (Figure 5D). T2* depends on the myelin [48, 49] and iron content [22, 30, 39, 48]. However, T2′, as compared to T2*, is thought to be less influenced by changes of the myelin fraction [48, 49], thus representing the more sensitive parameter for assessing the iron content. A previous study by Siemonsen et al. [50], investigating age-related changes of the parameters T2, T2* and T2′ in the cerebral white matter (WM) and deep GM in healthy volunteers, found a significant negative association between T2′ and age in the basal ganglia [50]. T2 values correlated positively with age, both in WM and in the deep grey nuclei [50]. T2* values were found to correlate negatively with age in deep GM structures, whereas in WM a positive correlation between T2* and age was detected. In contrast to this previous study, our study investigated associations between qMRI parameters in cortical GM and age, applying surface-based analysis techniques. The age-associated T2′ decrease in GM and T2 increase in WM and GM that were found in the previous study [50] are in line with our results, reflecting iron deposition and demyelination with increasing age. Furthermore, both studies indicate that cerebral T2* values seem to be influenced in a bidirectional manner during aging (Figure 5D). A potential explanation for this finding might be the presence of different microstructural processes taking place with a spatially heterogeneous pattern (Figure 5D), including both processes which increase T2* via inherent spin-spin effects (for example T2 increase attributable to demyelination) and processes which decrease T2* (such as iron deposition) (Figure 5D).

Global cortical PD values did not show a significant correlation with age (Figure 1B). Only in the temporal and occipital lobes a significant positive association with age could be detected (Figure 5B). However, partial volume effects might contribute to these results. PD quantifies free water in tissue [22, 51, 52] and therefore might be sensitive to age-related changes of the water-content. Since T1 is affected by the water and iron content [20, 53–57], T1 might be better suited to assess age-related tissue remodeling than PD. In summary, given the lack of a significant relationship of global T2* and global PD values with age, these parameters seem to be less sensitive to age-related changes of cortical microstructure than other qMRI parameters.

Global cortical T1 values were negatively correlated with age (Figure 1A). Similarly, the vertex-wise analysis, in consistence with the analysis based on cortical subregions, revealed negative relationships with age, mainly in frontal, parietal and (upper) temporal regions (Figures 2, 5A). These results are in line with the findings of previous studies investigating age-related changes of cortical T1 relaxation time, which described a decrease of T1 values between the 3^rd^ and the 8^th^ life decade [23–26]. Furthermore, a recent study investigating longitudinal changes of cortical T1 relaxation time in elderly subjects over seven years found a similar regional pattern of cortical T1 decrease, in particular for the frontal and the temporal cortex [20]. T1 is commonly considered a marker for the degree of myelination and for the proportion of macromolecules [21, 22, 53, 58–60], with an increasing myelin fraction typically leading to a decrease of T1 [22, 60, 61]. Since a decrease of overall myelination has been demonstrated with increasing age [16], changes of myelination may not be assumed to be responsible for the observed negative relationship between cortical T1 and age. Since T1 also depends on the water and iron content in tissue [20, 53–57], both a decreasing water fraction (induced by a regression of dendrites and a relative enhancement of the neuronal density [17, 20], and an increasing iron deposition (which takes place with progressing age [18, 19, 27]) may yield T1 shortening [20, 27, 30, 54]. The lack of a significant correlation between global cortical T1 values and cortical thickness may suggest that among the mechanisms driving age-related decreases of cortical T1 values, an increasing iron deposition is more relevant than a reducing tissue water fraction, since the latter is believed to be associated with a regression of dentrites [17, 20] and, accordingly, potentially with atrophy. By comparison with the T2 results (Figure 3A) it becomes evident that demyelination is likewise a prominent process associated with aging, which, however, might affect cortical T1 values less than microstructural changes of the iron or water proportions.

Areas with significant cortical thinning associated with higher age mainly involved the frontal cortex as well as some parts of the temporoparietal cortex (Figures 4, 5F, Supplementary Figure 2). This pattern matches well with the distribution of age-related cortical atrophy as described in the literature [62–64]. In general, age-related changes of qMRI parameters in the present study showed a larger extension across the cortex than the clusters observed for the cortical thickness with only partial overlap (Figures 2–4). Consequently, age-related qMRI parameter changes cannot primarily be attributed to cortical atrophy. While partial volume effects with CSF resulting from cortical atrophy could in theory partly contribute to increases of cortical PD, as well as increases of T2 and T2*, such contribution would not explain the observed decreases of T1, T2* and T2′ values, as partial volume effects from CSF would rather increase these parameters. It should be noted that the applied surface-based techniques are advantageous for the analysis of the thin cortical layer [65]. Since these methods are based on an exact definition of the white matter/cortex and cortex/CSF boundaries, respecting the folded cortical topology, surface-based analysis techniques can be assumed to potentially reduce partial volume effects as compared to other methods (such as the usage of cortical masks). Furthermore, the boundary-based co-registration approach used for this study allows for a robust alignment of the pial and white matter surfaces of different datasets [66], thus being relatively resistant to co-registration inaccuracies and resulting partial volume effects at tissue boundaries. Still, partial volume effects can only be reduced but not wholly excluded. Especially in cases where the individual standard deviation across the cortex for a given parameter deviates considerably from the mean standard deviation across the group (Supplementary Figure 1), an influence of partial volume effects might play a role. However, for the reasons discussed in detail above, it can be assumed that partial volume effects are not the underlying cause of the main findings of this study.

In summary, the results of the study presented here suggest that global iron deposition is the most relevant age-related microstructural cortical change, since quantitative T2′ shows the strongest correlation with age and the most widespread age-related changes across the cerebral cortex in comparison to other qMRI parameters (Figure 3B). Demyelination in higher age, as reflected by a positive correlation between T2 and age, might be most prominent in frontoparietal and upper temporal regions. Furthermore, the negative correlation between T1 and age in the same regions indicates microstructural changes affecting T1 beyond the more widespread iron deposition such as a decreasing water fraction caused by a regression of the dendrites, resulting in a relative increase of the neuronal density [17, 20]. PET research has revealed a strong age-related decrease in aerobic glycolysis and glucose consumption in frontal, parietal and temporal regions during adulthood [29]. This reduction of oxidative metabolism might affect energy-demanding repair processes and increase oxidative stress. The presented results indicate that these metabolic changes might be associated with demyelination and a regression of the dendrites, resulting in changes in water content, while global iron deposition might be caused by independent global metabolic processes.

### Limitations

This study has several limitations. First, the investigated sample size is relatively small. Second, since this is a cross-sectional study, longitudinal assessment of age-related changes of cortical qMRI parameters in individual subjects, or on a group level was beyond the scope of the study. Such analysis would allow for investigating the temporal dynamics of each parameter change (e.g. via determining an annual rate of change), thus providing a deeper understanding of how different microstructural changes underlying cortical aging evolve over time.

## CONCLUSIONS

Multiparametric qMRI allows for the characterization of cortical aging, demonstrating parameter-specific patterns and indicating that distinct biochemical processes on the microstructural level might dominate in certain cortical areas. In particular, demyelination and a shrinkage of the water fraction might predominantly affect frontal and some parietotemporal regions and might be linked to metabolic changes in these areas that had been observed in previous studies. In contrast, cortical iron deposition during aging appears to be a more prominent and global process. Therefore, especially quantitative T2′ values can be expected to be a promising imaging biomarker of cortical age-related remodeling and appear to be more sensitive to global cortical iron deposition than quantitative T2* values, which are not corrected for spin-spin effects: the effect of iron deposition on T2* may be masked by cortical microstructural processes leading to a prolongation of the cortical T2 relaxation time such as demyelination. A confirmation of our results in larger subject samples using a longitudinal design would be of interest.

## MATERIALS AND METHODS

### Study participants

The investigation was approved by the institutional ethics committee and performed according to the principles formulated in the declaration of Helsinki. 40 subjects (age range 19-71 years) who reported good health during anamnesis were recruited. Exclusion criteria were: pre-existing psychiatric or neurological disorders, uncontrolled arterial hypertension or diabetes mellitus and MRI contraindications. Medical history was carefully assessed, in order to exclude these medical conditions. The MP-RAGE datasets and the T2-weighted datasets (TE=86 ms) that had been derived as described in the sections below were inspected by an experienced neurologist to exclude the presence of cerebral pathologies. While leukoaraiosis was regarded as a normal process that occurs during aging [50], other pathological changes (such as for example a tumor or inflammatory lesions) were not observed. All participants gave written informed consent. The participants and the obtained data partly overlap with the healthy control cohorts of previous studies assessing structural changes in clinically isolated syndrome / RRMS [67, 68] while the present study investigates age-related cortical remodeling.

### MRI acquisition

Data were obtained using a 3 Tesla whole body Magnetom TRIO MR scanner (Siemens Healthineers, Erlangen), using a body coil for radio frequency (RF) transmission and a phased-array head coil with eight channels for signal reception.

Voxel-wise measurement of the T2 relaxation-time was based on the acquisition of four fast spin echo (FSE) datasets with different TE. In detail, the acquisition parameters were: field of view (FOV) = 240x180 mm^2^, 40 axial slices, slice thickness = 2 mm, inter-slice gap = 1 mm, resolution = 1.25x1.25 mm², TE= [17,86,103,120] ms, TR = 8 s, band width (BW) = 100 Hz/Pixel, refocusing angle = 180°, turbo factor = 11, echo spacing = 17.1 ms, total duration = 8:08 min for all four data sets.

Mapping of both T2* and B0 (for correcting T1 data) were based on the acquisition of eight multiple-echo gradient echo (GE) datasets with export of phase and modulus data: FOV = 240x180 mm^2^, 40 axial slices, slice thickness = 2 mm, inter-slice gap = 1 mm, resolution = 1.25 x 1.25 mm², TE = [10,16,22,28,34,40,46,52] ms, TR = 2400 ms, excitation angle (α) = 30°, BW = 299Hz/Pixel, duration = 5:46 min.

For motion correction, the acquisition was repeated twice with reduced spatial resolution (and therefore reduced k-space coverage) in phase encoding direction, covering only the central 50% (duration = 3:07 min) and 25% (duration = 1:41 min) of k-space.

To map the profile of the transmitted RF field B1, two GE datasets were acquired, one with a preceding RF pulse followed by a gradient spoiler pulse. This causes a reduction of the longitudinal magnetization. B1 is calculated by comparing the signal levels in both datasets as described in the literature [69]. The parameters were: FOV = 256x224x160 mm³, isotropic resolution = 4 mm, TE = 5 ms, TR = 11 ms, α = 11°, BW = 260 Hz/Pixel, duration = 0:53 min.

For mapping of T1 and PD, two different excitation angles were applied to acquire a PD- and a T1-weighted spoiled GE dataset. A fast low angle shot (FLASH) echo-planar imaging (EPI) hybrid readout was used to increase the signal-to-noise ratio. The acquisition parameters were: FOV = 256x224x160 mm³, isotropic resolution = 1 mm, TE = 6.7 ms, TR = 16.4 ms, α1 = 4°, α2 =24°, BW = 222 Hz/Pixel, duration: 9:48 min for both data sets. For correcting the PD-weighted data set for T2*-effects which is required to obtain unbiased PD values, the acquisition of two GE-datasets with a TE-difference of 6.7 ms was performed, as described previously [51, 70]. The FoV was the same as for B1 mapping and the isotropic resolution was 2 mm.

The total acquisition time for all datasets was 34:23 min.

### Analysis

Analyses were conducted using MatLab (MathWorks) and the toolboxes FSL [71] (FMRIB) and FreeSurfer [72] (Athinoula A. Martinos Center for Biomedical Imaging).

The variable flip angle method was used for voxel-wise mapping of the T1-relaxation time [73]. Given that α_1/2_ are the applied flip angles and S_1/2_ the signal intensities in the datasets with PD/T1 weighting, T1 is measured by plotting S_i_/sin(α_i_) versus S_i_/tan(α_i_). This plot exhibits a linear dependence with the slope exp(-TR/T1) and allows for the determination of T1. To correct T1 data for B1 effects, B1-inhomogeneities were assessed as described above and in the literature [69], using only B1-corrected excitation angles in the plot described above. Furthermore, the T1 mapping algorithm comprised compensation for B0-distortions [74]. These were measured using the phase data acquired for T2*-mapping and applying FSL PRELUDE and FUGUE. Additionally, corrections for the effect of insufficient spoiling of transverse magnetization were carried out as described previously [75].

Voxel-wise mapping of PD was performed by eliminating different effects from PD-weighted data. In particular, we compensated for 1) residual T1- and T2*-weighting, 2) non-uniformities of B1 and 3) any bias imposed by the sensitivity profile of the receive coil. The complete procedure has been described in the literature [76].

T2- and T2*-mapping was performed via mono-exponential fitting of the signal in the series of FSE (T2) or GE (T2*) datasets obtained with multiple TE. The algorithms included correction of T2*-weighted data for motion [77], replacing motion-affected lines in k-space by the respective lines that had been acquired in the additional data sets with reduced k-space sampling. The effects of B0 distortions were eliminated from the T2*-data [78] and the effects of stimulated and secondary echoes from the T2-maps, as reported previously [79]. T2* maps were co-registered to the T2 maps (six degrees of freedom). T2′ maps were derived from T2* and T2 maps according to [80]: 1/T2′=1/T2*−1/T2.

Synthetic MP-RAGE anatomies with mixed T1/PD-weighting were derived from the T1 maps as reported previously in the literature [74, 81]. Virtual acquisition parameters for these synthetic MP-RAGE data were: FOV = 256x224x160 mm³, isotropic resolution = 1 mm, TR = 1.9 s, TI = 900 ms, α = 9°.

The “recon-all” stream implemented in the FreeSurfer toolbox [72] was applied to the synthetic MP-RAGE data for cortical segmentation, for detection of the pial and WM/cortex surfaces and for vertex-wise measurement of the cortical thickness. The total intracranial volume (TIV) was determined with FreeSurfer from the synthetic MP-RAGE data.

While the T1- and PD-maps were already in alignment with the synthetic datasets (which had been derived from the T1 maps), co-registration matrices were created between T2-maps and the anatomies using BBREGISTER for boundary-based registration. Cortical T1-, PD-, T2-, T2*- and T2′-values were sampled with mri_vol2surf and stored in surface datasets. To this aim, the matrices describing the registration of the T2-maps to the MP-RAGE datasets were used to read T2, T2* and T2′ values, since these parameter maps had the same orientation. The qMRI values were sampled without changing the resolution of the respective maps.

The relationship of cortical MRI parameters and age was analyzed with three different approaches: 1) for average values across the cortex, 2) assessing correlations vertex-wise and 3) for different cortical regions of interest (lobes). The respective analyses were performed as follows:

1. To determine and further analyze global cortical T1, PD, T2, T2*, T2′ and cortical thickness values, average values (and SDs) across all cortical vertices were obtained for each subject as described previously [68]. We tested for (partial) Pearson correlations between average cortical MRI parameters and age with and without correcting for TIV and sex as nuisance variables.
2. After normalizing (“FSAVERAGE” space) and smoothing the surface-datasets (Gaussian kernel, full width at half maximum = 1 cm), surface-based tests were performed to identify local areas of significant correlations between age and those MRI parameters, for which a significant correlation with age had been observed on a global level. Monte Carlo simulations were calculated to identify clusters indicating significant correlations and to correct for multiple comparisons. Since it was intended to visualize the spatial cortical distributions for parameters correlating globally with age, all vertices with significant p-values (< 0.05) were included in this simulation.
3. For the analysis of MRI parameters in the different cortical lobes a similar approach was followed as described in a previous study [67]. In summary, the cortical atlas “PALS_B12_Lobes” was co-registered to the participants’ surfaces with the tool “mri_surf2surf. Subsequently, average qMRI and thickness values were extracted for all participants from the frontal, temporal, parietal and occipital cortical regions of interest. Since surface-based analysis demonstrated mostly symmetric distributions across both hemispheres, parameter values averaged across both hemispheres were used to test for Pearson correlations with age for the cortical regions. Furthermore, for each cortical area, tests for Pearson correlations of the qMRI parameters and the cortical thickness in the respective region were performed, using the TIV as a covariate. P values below 0.05 were considered significant for all statistical tests.

## Supplementary Material

Supplementary Figures
