# Supplementary material for: Cortical aging – new insights with multiparametric quantitative MRI
Source: Aging (Albany NY). 2020 Aug 27;12(16):16195–210. doi: 10.18632/aging.103629 (PMC7485732; doi:10.18632/aging.103629)
Supplement: Supplementary Figures [file aging-12-103629-s001..pdf]

SUPPLEMENTARY FIGURES

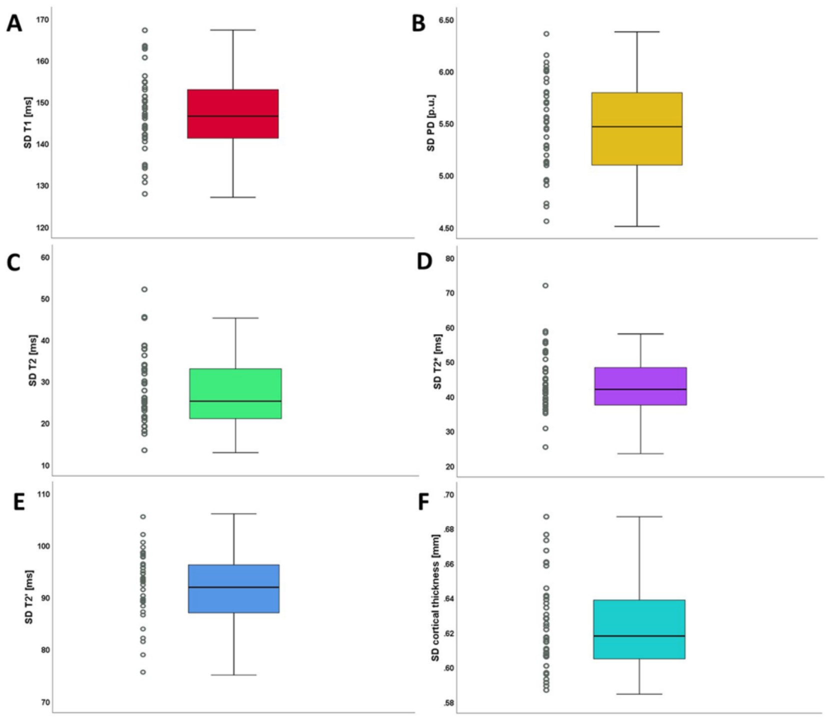

Supplementary Figure 1. Standard deviations of cortical MRI parameters across the cortex. (**A**) standard deviation of T1; (**B**) standard deviation of PD; (**C**–**E**) standard deviations of T2, T2* and T2'; (**F**) standard deviation of the cortical thickness. In each panel, each data point on the left reflects the individual standard deviation across the cortex for a single participant for the respective cortical parameter. The boxplots on the right illustrate the median across the group of the standard deviations across the cortex, along with the 1st and the 3rd quartile as well as the minimum and the maximum value. Circles with a distance of more than 1.5 box lengths from the median represent outliers which were not considered for calculation of the minimum and the maximum. SD: standard deviation; ms: milliseconds; p.u.: percentage units; mm: millimeters.

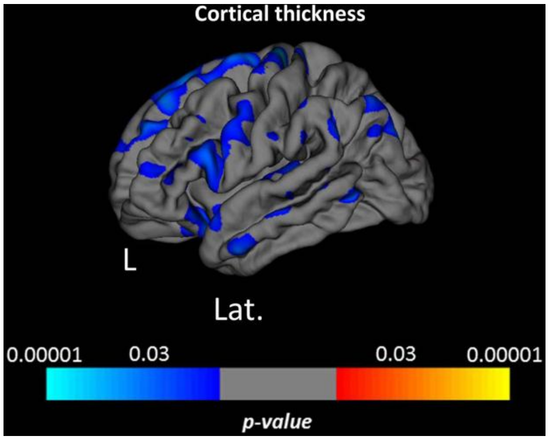

Supplementary Figure 2. Cortical clusters indicating a significant association between age and cortical thickness before correction for multiple comparisons for the left lateral hemisphere. The scale bar displays the level of significance. Cold colours demonstrate a negative association with age in the respective regions. L: left; Lat.: lateral.
